# Increased circulating Tfh to Tfr ratio in chronic renal allograft dysfunction: a pilot study

**DOI:** 10.1186/s12865-019-0308-x

**Published:** 2019-08-05

**Authors:** Lin Yan, Yamei Li, Yi Li, Xiaojuan Wu, Xianding Wang, Lanlan Wang, Yunying Shi, Jiangtao Tang

**Affiliations:** 1 Department of Laboratory Medicine, West China Hospital, Sichuan University, No.37 Guoxue Xiang, Wuhou District, Chengdu, Sichuan China; 2 Department of Urology, West China Hospital, Sichuan University, Chengdu, China; 3 Department of Nephrology, West China Hospital, Sichuan University, Chengdu, China

**Keywords:** T follicular helper cells, T follicular regulatory cells, cTfh to cTfr ratio, CXCL13, Chronic renal allograft dysfunction

## Abstract

**Background:**

T follicular helper (Tfh) cells play a control role in contribution of B cell differentiation and antibody production. T follicular regulatory (Tfr) cells inhibit Tfh-B cell interaction.

**Methods:**

To identify whether circulating Tfh (cTfh) and Tfr (cTfr) cells contribute to chronic renal allograft dysfunction (CAD), 67 kidney transplant recipients (34 recipients with CAD, 33 recipients with stable function) were enrolled. The frequency of cTfh and cTfr cells, the level of serum CXCL13 were measured.

**Results:**

The frequency of cTfr cells in CAD group was significantly lower than that in stable group (0.31% vs 0.68%, *P* = 0.002). The cTfh to cTfr ratio in CAD group was significantly higher than that in stable group (55.4 vs 25.3, *P* = 0.013). Serum CXCL13 in CAD group was significantly higher than stable group (30.4 vs 21.9 ng/ml, *P* = 0.025). After linear regression analysis, the cTfh to cTfr ratio was an independent risk factor for estimated glomerular filtration rate (eGFR) in recipients (standardized coefficient = − 0.420, *P* = 0.012). After logistic regression analysis, the cTfh to cTfr ratio was an independent risk factor for CAD (OR = 1.043, 95%CI = 1.004–1.085, *P* = 0.031).

**Conclusion:**

The imbalance between cTfh and cTfr cells contribute to the development of CAD.

**Electronic supplementary material:**

The online version of this article (10.1186/s12865-019-0308-x) contains supplementary material, which is available to authorized users.

## Background

The risk of acute rejection after kidney transplantation has been decreased with the development of immunosuppressant and transplant technique, while chronic renal allograft dysfunction (CAD) is still the main threat for long-term allograft survival rates. Antibody-mediated injury or rejection is the leading cause of late kidney allograft dysfunction [1, 2]. Donor-specific antibodies (DSA) could identify patients at high risk for kidney allograft loss [3, 4]. Avoiding the influence of humoral immune factors on allograft function could decline the risk of CAD.

The production of high affinity antibody in germinal center (GC) requires the help of T follicular helper (Tfh) cells [5]. Tfh cells in lymph node highly express C-X-C chemokine receptor 5 (CXCR5), programmed death 1 (PD-1) and inducible co-stimulator (ICOS) [6]. Tfh cells migrate into germinal centers via gradients of C-X-C chemokine ligand 3 (CXCL13) and initiate B cells to undergo proliferation, differentiation and somatic hypermutation [6]. Tfh differentiation relies on expression of B cell lymphoma 6 (Bcl-6), which promote ICOS and PD-1 expression [6]. It has been demonstrated that signal transducer and activator of transcription 3 (STAT3) is required for the differentiation of Tfh cells through the induction of Bcl-6 [7–10]. Bcl-6 within Tfh cells is negatively regulated by signal transducer and activator of transcription 5 (STAT5), which inhibit Tfh differentiation through increasing the expression of B lymphocyte-induced maturation protein 1 (Blimp-1) [11, 12].

Tfh cells could also migrate to the circulation as circulating Tfh (cTfh) cells. These circulating cells express lower amounts of the Tfh markers ICOS, CXCR5, PD-1 and Bcl-6 than their germinal center counterparts and respond to CXCL13 chemokine gradients, moving back to a secondary lymphoid organ germinal center, where they may be involved in germinal center formation [6]. Whether serum CXCL13 level is associated with cTfh cells in kidney transplantation recipients is not clear.

A recently described T follicular regulatory (Tfr) cells has revealed a new means by which the GC reaction is controlled [13]. Tfr cells express high levels of CXCR5, which directs them to the GC to inhibit the interaction of Tfh and B cells. Tfr cells differ from Tfh cells by expressing foxkhead box P3 (FoxP3) and Blimp-1 [13]. The dynamic proportions of Tfh and Tfr cells precede the increase in GC-B cells and antibody production [13]. Whether the circulating Tfr (cTfr) cells or the cTfh to cTfr ratio is associated with CAD is not clear.

It is well-known that transforming growth factor beta (TGF-β) plays a critical role in immune regulation, particularly in generation, function and stabilization of regulatory T cells (Tregs) [14]. TGF-β could also regulate the development of Tfh and Tfr cells. Schmitt et al. found that TGFβ could promote human Tfh cells differentiation through STAT3/STAT4-mediated signal pathway [8]. TGF-β neutralization could partially weaken the inhibitory effect of Tfr cells on the proliferation and differentiation of Tfh cells and B cells [15]. TGF-β could also contribute to the development of Tfr cells through promoting the generation and activation of Treg cells [16]. Therefore, it is needed to identify that whether the role of serum TGF-β in kidney transplant recipients favoring immune regulation or immune reactivation.

It is not clear that whether the cTfh and cTfr cells, the cTfh to cTfr ratio, the expression of STAT3/STAT5 on CD4^+^CXCR5^+^ cells, serum CXCL13 and TGF-β are correlated with CAD in kidney transplant recipients. The aim of this study was to identity the possible association between these immune parameters and CAD, and further probe into the underlying mechanism of CAD.

## Methods

### Patients

The present study is a cross-sectional pilot study. Eighty-two kidney transplant recipients receiving living donor kidney in West China Hospital of Sichuan University were enrolled in this study from May 2016 to May 2017. All of the recipients with infection or autoimmune diseases at the time of analysis were excluded from this study. All of the heparin-anticoagulated whole blood from these patients were performed flow cytometry. Excluded the number of acquired target cell subsets less than 100 cells, 67 recipients were eventually included in the following analysis. Chronic allograft dysfunction was defined as estimated glomerular filtration rate (eGFR) < 60 ml/min/1.73m^2^ after 3 months of transplantation [17, 18]. Within the 67 recipients, 34 recipients suffered from CAD (defined as CAD group) and 33 had stable renal function (defined as stable group). Among the 34 recipients with CAD, 21 recipients had undergone biopsy. According to Banff-2015 [19], 13 recipients were defined as biopsy-proven rejection (BPR) with 11 antibody-mediated rejection (ABMR) and 2 T cell-mediated rejection (TCMR), 9 recipients were defined as non-rejection (4 interstitial fibrosis tubular atrophy, 3 transplant glomerulonephropathy, 1 BK virus nephropathy, 1 recurrent glomerulonephropathy). Only 6 of all recipients with CAD got DSA detection with the results of 5 positive and 1 negative. Fifty recipients of all got panel reactive antibodies (PRA) detection with the results of 35 positive and 15 negative. BPR, non-rejection, DSA, PRA were used for sub-group analysis.

### Immunosuppressive regimen

All of 67 patients received basiliximab as prophylactic therapy. Forty-eight recipients received tacrolimus-based triple immunosuppressant regimen (tacrolimus + mycophenolate mofetil + prednisone); 12 recipients received sirolimus-based triple immunosuppressant regimen (sirolimus + mycophenolate mofetil + prednisone); 2 recipients received cyclosporine A-based triple immunosuppressant regimen (cyclosporine A + mycophenolate mofetil + prednisone); 5 recipients received the combined tacrolimus-minimized and sirolimus immunosuppressant regimen (tacrolimus + sirolimus + mycophenolate mofetil + prednisone). Tacrolimus dose was administered at 1.0–1.5 mg bid. The tacrolimus-minimized regimen was 0.5 mg bid. The dose of sirolimus was 1.0 mg bid. Cyclosporine A was administered at 50–75 mg bid. Mycophenolate mofetil (MMF) was administered at 750 mg bid. The maintenance dose of prednisone was 5 mg or 10 mg qd.

### Flow cytometry

To determine the percentage of T cell subsets, heparin-anticoagulated whole blood were collected and stained with CD3-PerCP (BD Bioscience, New Jersey, US), CD4-FITC (BD Bioscience, New Jersey, US), CXCR5-APC (Biolegand, California, US), PD-1-PE (eBioscience, California, US), ICOS-PE (eBioscience, California, US) and CD25-APC (BD Bioscience, New Jersey, US). After fixed and permeabilized, samples were stained with FoxP3-PE (BD Bioscience, New Jersey, US), p-STAT3-PE (BD Bioscience, New Jersey, US), p-STAT5-PE (BD Bioscience, New Jersey, US) and p-STAT4-PE (BD Bioscience, New Jersey, US). After stimulation with phorbol 12-myristate 13-acetate (PMA) (50 ng/ml) (Sigma-Aldrich, US), ionomycin (1 μg/ml) (Sigma-Aldrich, US), and Golgi stop (BD Bioscience, New Jersey, US) for 5 h, the fixation and permeablication were performed. Then samples were stained with IL-21-PE (BD Bioscience, New Jersey, US). Samples were measured with FACS Canto II (BD Biosciences, New Jersey, US). Gating strategy used for the analysis of all immune parameters was shown in Additional file 1.

### Bio-plex

Serum samples were collected and stored at − 80 °C freezer until analysis. Human Premixed Multi-Analyte Kit was purchased from R&D Systems (Minneapolis, Minnesota, USA). Serum CXCL13 and TGF-β were measured by Bio-Plex® suspension array system (Bio-Rad Laboratories Inc., California, USA). All samples were measured in duplicate. Four serum samples were excluded from this analysis as the volume were not enough for analysis. Two CXCL13 detection results were also excluded as they were reported with warning after bio-plex analysis. Eventually, 61 results of CXCL13 and 63 results of TGF-β were included in the following analysis.

### Laboratory assays

Serum creatinine (Scr) was measured by picric acid method (Roche Diagnostics, Mannheim, Germany). The eGFR was calculated using the Modification of Diet in Renal Disease formula which was adjusted to Chinese [20]: eGFR (ml/min/1.73m^2^) = 186 × Scr (mg/dl)^-1.154^ × age^-0.203^ × (0.742 if female) × 1.233.

### Statistics analysis

Statistical analysis and graphics were performed using SPSS 21.0 (SPSSInc, Chicago, IL, US) and GraphPad Prism version 5.01 (GraphPad, Inc., La Jolla, CA). The Mann-Whitney U-test was utilized to identify differences in phenotype between different groups. Logistic regression was performed to assess the independent associations of immune-associated parameters, other clinical variables with CAD. Linear regression was used to assess the independent associations of immune-associated parameters, other clinical variables with eGFR. Chi-square test and Mann-Whitney U-test were used to compare the percentage of recipients with CAD between groups classified based on cTfh to cTfr ratio. Spearman correlation analysis was performed to assess the association between CXCL13 or TGF-β and the phenotype of cTfh or cTfr cells. A two-sided *p*-value ≤0.05 was considered significant.

## Results

### Demographic and clinical characteristics

This study eventually enrolled in a total of 67 candidates. Within the 67 kidney transplant recipients, 34 recipients suffered chronic allograft dysfunction and 33 maintained stable renal function. The demographic and clinical characteristics in the present study were described in Table 1. There were no significant differences in age, gender, pre-PRA level, HLA mismatch and transplant duration time between CAD group and stable group. Sixty-two recipients received tacrolimus, sirolimus or cyclosporine A based immunosuppressive regiment combined with prednisone and mycophenolate mofetil. Five recipients received combined-use of calcineurin inhibitor (CNI) and sirolimus in CAD group. The use of immunosuppressant was statistically different between CAD group and stable group (*P* = 0.045). The eGFR level was significantly different between CAD group and stable group (median value: 33.2 vs 74.1 ml/min/1.73m^2^, respectively, *P* < 0.001).

**Table 1:** Demographic and clinical characteristics

	CAD group	Stable group	*P*-value
	*N* = 34	*N* = 33	
Age (range), years	44 (22–53)	41 (26–53)	0.213
Male, percentage	28, 82%	24, 73%	0.352
Pre-PRA (range), %	0 (0–24.1)	0 (0–26.8)	0.111
HLA mismatch (range)	4 (2–7)	4 (0–6)	0.164
Transplant Duration (range), months	55 (6–132)	36 (6–108)	0.089
Immunosuppressant			**0.045**
--Tacrolimus	--21	--27	
--Sirolimus	--7	--5	
--Cyclosporine A	--1	--1	
--CNI + Sirolimus	--5	--0	
eGFR (range), ml/min/1.73m^2^	33.2 (4.8–58.9)	74.1 (60.9–121.6)	**< 0.001**

*P*-value < 0.05 was shown in bold. Data were shown as Median (Range) or Number, percentage

*CAD* chronic allograft dysfunction, *PRA* panel reactive antibodies, *CNI* calcineurin inhibitor

### Decreased frequency of cTfr cells and increased cTfh to cTfr ratio in CAD group

**Fig. 1:**
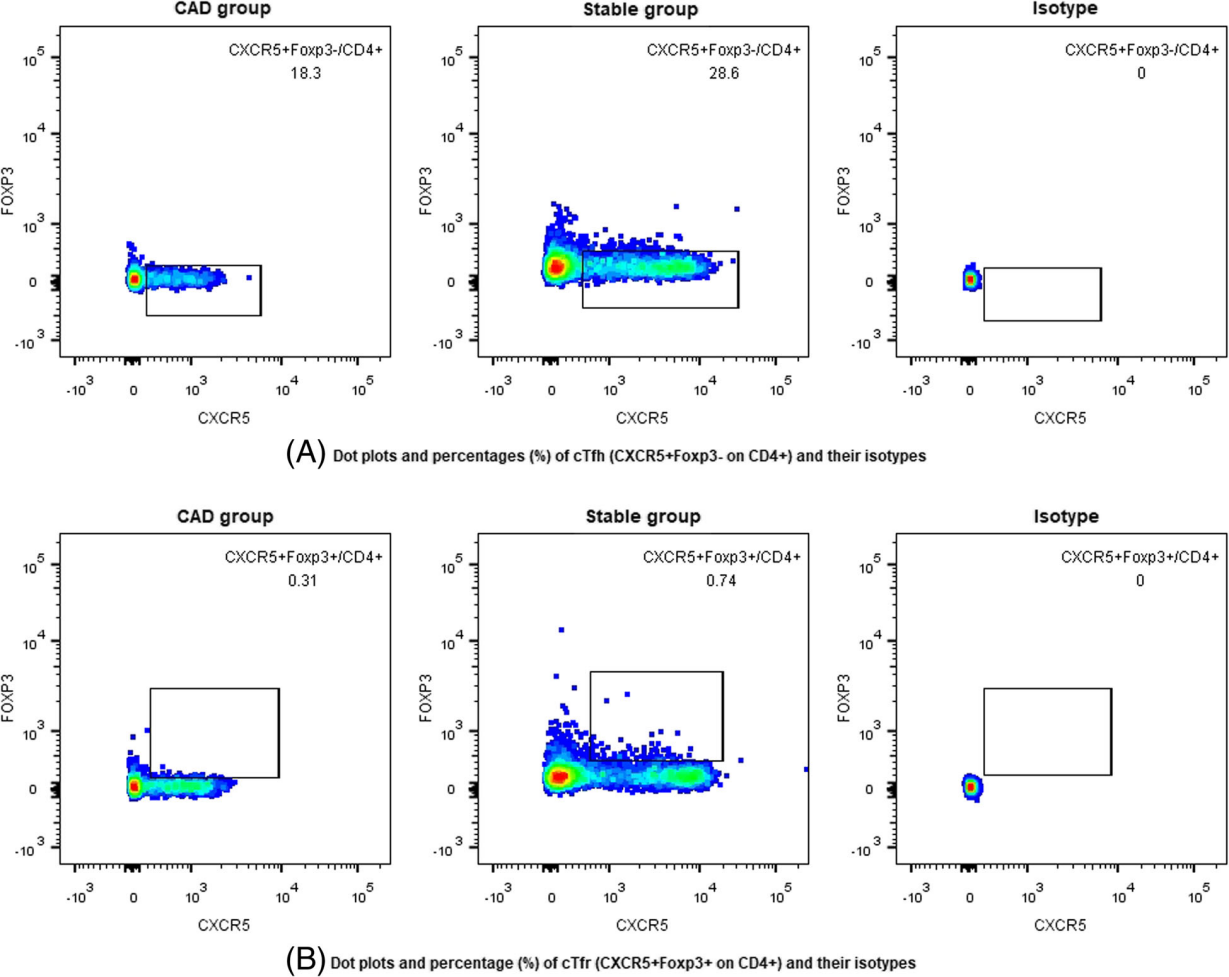
Dot plots of cTfh, cTfr cells and their isotypes between CAD group and stable group. **a** Representative of dot plots and percentage (%) of cTfh (CXCR5^+^Foxp3^−^/CD4^+^) cells and their isotypes between CAD group and stable group; **b** Representative of dot plots and percentage (%) of cTfr (CXCR5^+^Foxp3^+^/CD4^+^) cells and their isotypes between CAD group and stable group

**Fig. 2:**
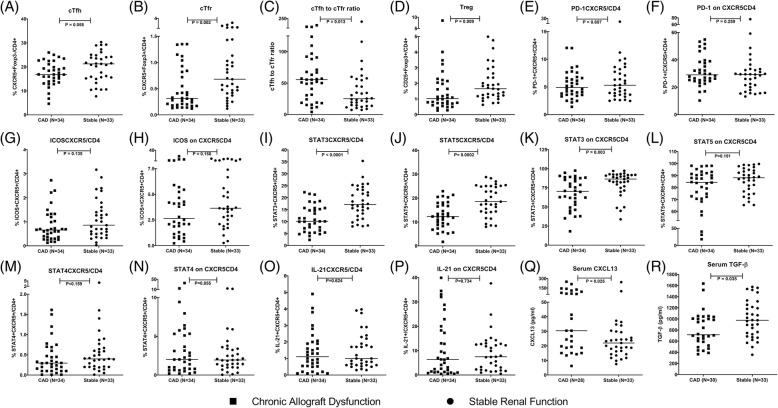
The frequency of cTfh and cTfr cells and the level of associated factors between CAD group and stable group**.** Squares refer to chronic allograft dysfunction (CAD) group, cycles refer to stable renal function group; **a** cTfh: CXCR5^+^Foxp3^−^ on CD4^+^ cells; **b** cTfr: CXCR5^+^Foxp3^+^ on CD4^+^ cells; **c** cTfh to cTfr ratio; **d** Tregs: CD25^+^Foxp3^+^ on CD4^+^ cells; **e** CXCR5^+^PD-1^+^ on CD4^+^ cells; **f** PD-1^+^ on CXCR5^+^CD4^+^ cells; **g** CXCR5^+^ICOS^+^ on CD4^+^ cells; **h** ICOS^+^ on CXCR5^+^CD4^+^; **i** CXCR5^+^STAT3^+^ on CD4^+^ cells; **j** CXCR5^+^STAT5^+^ on CD4^+^ cells; **k** STAT3^+^ on CXCR5^+^CD4^+^ cells; **l** STAT5^+^ on CXCR5^+^CD4^+^ cells; **m** CXCR5^+^STAT4^+^ on CD4^+^ cells; **n** STAT4^+^ on CXCR5^+^CD4^+^ cells; **o** CXCR5^+^IL-21^+^ on CD4^+^ cells; **p** IL-21^+^ on CXCR5^+^CD4^+^ cells; **q** The serum level of CXCL13; **r** The serum level of TGF-β

The frequency of CXCR5^+^ on CD4^+^ cells was significantly lower in CAD group compared to stable group (17.3% vs 22.2%, *P* = 0.035). The frequency of cTfh cells (CXCR5^+^Foxp3^−^ on CD4^+^) had a lower trend in CAD group compared to stable group (16.8% vs 21.2%, *P* = 0.058, Figs. 1a, 2a). The frequency of cTfr (CXCR5^+^Foxp3^+^ on CD4^+^) cells in CAD group was observed significantly lower than that in stable group (0.31% vs 0.68%, *P* = 0.002, Figs. 1b, 2b). The cTfh to cTfr ratio was significantly higher in CAD group compared to stable group (55.4 vs 25.3, *P* = 0.013, Fig. 2c). Tregs (CD25^+^Foxp3^+^ on CD4^+^) showed the same trend as cTfr cells (1.03% vs 1.66%, *P* = 0.009, Fig. 2d).

The differences of the frequency of CXCR5^+^PD-1^+^ on CD4^+^ cells or PD-1 expression on CD4^+^CXCR5^+^ cells were not significant between CAD group and stable group (4.9% vs 5.3%, *P* = 0.607, Fig. 2e; 29.3% vs 29.1%, *P* = 0.259, Fig. 2f, respectively). Neither as the frequency of CXCR5^+^ICOS^+^ on CD4^+^ cells or ICOS expression on CD4^+^CXCR5^+^ cells between CAD group and stable group (0.66% vs 0.86%, *P* = 0.135, Fig. 2g; 2.6% vs 3.6%, *P* = 0.158, Fig. 2h, respectively).

There was significantly lower frequency of CXCR5^+^STAT3^+^ on CD4^+^ or CXCR5^+^STAT5^+^ on CD4^+^ cells in CAD group than patients with stable renal function (10.1% vs 17.2%, *P* < 0.0001, Fig. 2i; 12.3% vs 18.5%, *P* = 0.0002, Fig. 2j). The STAT3 expression on CD4^+^CXCR5^+^ cells in CAD group was also significantly lower than that in stable group (70.3% vs 86.4%, *P* = 0.003, Fig. 2k), while the expression of STAT5 on CD4^+^CXCR5^+^ cells had no significant differences between CAD group and stable group (84.3% vs 88.1%, *P* = 0.151, Fig. 2l).

However, the frequency of CXCR5^+^STAT4^+^ on CD4^+^ and the expression of STAT4 on CD4^+^CXCR5^+^ cells had no significant differences between CAD group and stable group (0.29% vs 0.40%, *P* = 0.159, Fig. 2m; 2.0% vs 1.9%, *P* = 0.855, Fig. 2n, respectively). Neither as the frequency of CXCR5^+^IL-21^+^ on CD4^+^ cells or IL-21 expression on CD4^+^CXCR5^+^ cells between CAD group and stable group (1.11% vs 1.00%, *P* = 0.624, Fig. 2o; 6.3% vs 7.5%, *P* = 0.734, Fig. 2p, respectively).

### Increased serum CXCL13 and decreased serum TGF-β in CAD group

Serum CXCL13 median level were 21.9 (15.2–29.6) ng/ml in patients with stable renal function and 30.4 (18.7–86.9) ng/ml in patients with CAD. Serum CXCL13 was significantly higher in CAD group when compared to stable group (*P* = 0.025, Fig. 2q). The median level of serum TGF-β were 976 (704–1235) pg/ml in patients with stable renal function and 716 (572–1014) pg/ml in patients with CAD. It was significantly lower in CAD group compared to stable group (*P* = 0.035, Fig. 2r).

### The cTfh to cTfr ratio was an independent risk factor to renal function and CAD

In model 1, we assessed whether the association between immune parameters and eGFR remained independent of adjustment for age, gender, transplantation duration time, pre-PRA level, HLA mismatch and immunosuppressant. The cTfh to cTfr ratio, CXCR5^+^STAT3^+^ on CD4^+^ cells, CXCR5^+^STAT5^+^ on CD4^+^ cells, Tregs or CXCL13 was independent factor to eGFR (standardized coefficient = − 0.279, *P* = 0.030; standardized coefficient = 0.328, *P* = 0.007; standardized coefficient = 0.327, *P* = 0.008; standardized coefficient = 0.399, *P* = 0.001; standardized coefficient = − 0.380, *P* = 0.006, respectively). To assess whether these five parameters were independent of each other, second regression analysis was performed after including these five parameters in one multiple linear regression analysis. The cTfh to cTfr ratio was observed an independent risk factor to declined eGFR (standardized coefficient = − 0.420, *P* = 0.012, shown in Table 2).

**Table 2:** Multi-regression analysis

Model 1 Linear regression analysis for eGFR				
	Standardized coefficient			*P*-value
Gender	−0.207			0.110
Age	−0.193			0.225
Transplantation duration	−0.191			0.195
Pre-PRA	0.015			0.909
HLA mismatch	0.025			0.852
Immunosuppressant	0.094			0.548
cTfh to cTfr ratio	−0.420			**0.012**
CXCR5^+^STAT3^+^ on CD4^+^	−0.236			0.374
CXCR5^+^STAT5^+^ on CD4^+^	0.273			0.269
Tregs	0.101			0.533
CXCL13	−0.162			0.303
Model 2 Logistic regression analysis for CAD				
	OR	OR 95% CI		*P*-value
Gender	9.964	0.185	535.386	0.258
Age	0.993	0.838	1.176	0.932
Transplantation duration	1.042	1.007	1.078	**0.018**
Pre-PRA	0.912	0.770	1.080	0.284
HLA mismatch	0.827	0.397	1.722	0.612
Immunosuppressant	0.541	0.106	2.750	0.459
cTfh to cTfr ratio	1.043	1.004	1.085	**0.031**
CXCR5^+^STAT3^+^ on CD4^+^	1.093	0.733	1.628	0.663
CXCR5^+^STAT5^+^ on CD4^+^	0.760	0.492	1.174	0.216
Tregs	0.971	0.276	3.417	0.963
cTfr	3.304	0.029	375.073	0.621
CXCL13	1.023	0.968	1.082	0.415

Model 1. *P*-value for linear regression equation was 0.004 by multiple linear regression analysis including age, gender, transplantation duration, Pre-PRA, HLA mismatch, immunosuppressant, cTfh to cTfr ratio, CXCR5^+^STAT3^+^ on CD4^+^ cells, CXCR5^+^STAT5^+^ on CD4^+^ cells, Treg, CXCL13; Model 2. R square for logistic regression was 0.681 by multiple logistic regression analysis including age, gender, transplantation duration, Pre-PRA, HLA mismatch, immunosuppressant, cTfh to cTfr ratio, CXCR5^+^STAT3^+^ on CD4^+^ cells, CXCR5^+^STAT5^+^ on CD4^+^ cells, cTfr, Treg, CXCL13; *P*-value < 0.05 was shown in bold. *Pre-PRA* panel reactive antibodies prior to transplantation, *OR* odds ratio, *CI* confidence intervals

In model 2, we assessed whether the association between immune parameters and CAD remained independent of adjustment for age, gender, transplantation duration time, pre-PRA level, HLA mismatch and immunosuppressant. The cTfh to cTfr ratio, CXCR5^+^STAT3^+^ on CD4^+^ cells, CXCR5^+^STAT5^+^ on CD4^+^ cells, cTfr, Tregs or CXCL13 was independent factor to eGFR (standardized coefficient = 1.019, *P* = 0.024; standardized coefficient = 0.868, *P* = 0.002; standardized coefficient = 0.327, *P* = 0.008; standardized coefficient = 0.250, *P* = 0.033; standardized coefficient = 0.344, *P* = 0.007; standardized coefficient = 1.038, *P* = 0.031, respectively). Second regression analysis was also performed after including these six parameters in one multiple linear regression analysis. Transplantation duration and cTfh to cTfr ratio were independent risk factors to CAD (OR = 1.042, 95%CI 1.007–1.078, *P* = 0.018; OR = 1.043, 95%CI 1.004–1.085, *P* = 0.031, shown in Table 2).

### Stratified analysis of cTfh to cTfr ratio

Based on the quartile of cTfh to cTfr ratio, the kidney transplant recipients were classified into four groups, Group 1 (ratio ≤ 16), Group 2 (16 < ratio ≤ 35), Group 3 (35 < ratio ≤ 60), Group 4 (ratio > 60). Within Group 1, Group 2, Group 3 or Group 4, the percentage of recipients with CAD was 33.3, 33.3, 64.7, 70.6%, respectively. The composition ratio of recipients with stable renal function and CAD within these four groups was significantly different (*P* = 0.046) by Chi-square test. Through post-hoc test by Mann-Whitney U methods, the percentage of recipients with CAD in Group 4 was significantly higher than that in Group 1 and Group 2 (*P* = 0.038; *P* = 0.030, respectively, Table 3). No significant difference of the percentage of recipients with CAD between Group 1 and Group 2, Group 1 and Group3, Group 2 and Group 3, Group 3 and Group 4 was found (*P* = 1.000, *P* = 0.081, *P* = 0.067, *P* = 0.718, respectively).

**Table 3:** Stratified analysis of cTfh to cTfr ratio

		Stable group	CAD group	Total	*P*-value
Group 1	N	10	5	15	1.000 (Group 1 vs Group 2)
	Percentage	66.7%	33.3%		0.081 (Group 1 vs Group 3)
Group 2	N	12	6	18	**0.038** (Group 1 vs Group 4)
	Percentage	66.75	33.3%		0.067 (Group 2 vs Group 3)
Group 3	N	6	11	17	**0.030** (Group 2 vs Group 4)
	Percentage	35.3%	64.7%		0.718 (Group 3 vs Group 4)
Group 4	N	5	12	17	
	Percentage	29.4%	70.6%		
Total	N	33	34	67	
	Percentage	49.3%	50.7%		
*P*-value	**0.046**				

Group 1 (cTfh to cTfr ratio ≤ 16); Group 2 (16 < cTfh to cTfr ratio ≤ 35); Group 3 (35 < cTfh to cTfr ratio ≤ 60); Group 4 (cTfh to cTfr ratio > 60). *P*-value < 0.05 was shown in bold

### Correlation analysis of CXCL13 or TGF-β for cTfh or cTfr

After correlation analysis, a negative association between serum CXCL13 and frequency of CXCR5^+^ on CD4^+^ cells was observed in kidney transplant recipients (spearman *r* = − 0.332; *P* = 0.008, Table 4). The frequency of cTfh cells was also negatively correlated with CXCL13 (spearman *r* = − 0.312; *P* = 0.013, Table 4). No association between serum CXCL13 and cTfr cells was observed (spearman *r* = − 0.108; *P* = 0.435, Table 4). No association between serum TGF-β and cTfh, cTfr, CXCR5^+^STAT3^+^ on CD4^+^ cells, or Tregs was observed (Table 4).

**Table 4:** Correlation analysis of CXCL13 or TGF-β for cTfh or cTfr

		Spearman r	*P*-value
CXCL13	CXCR5^+^ on CD4^+^	−0.332	**0.008**
CXCL13	cTfh	−0.312	**0.013**
CXCL13	cTfr	−0.108	0.435
TGF-β	cTfh	0.158	0.209
TGF-β	cTfr	0.249	0.064
TGF-β	CXCR5^+^STAT3^+^ on CD4^+^	0.206	0.106
TGF-β	Tregs	0.068	0.596

*P*-value < 0.05 was shown in bold

### Sub-group analysis based on BPR, DSA and PRA

**Fig. 3:**
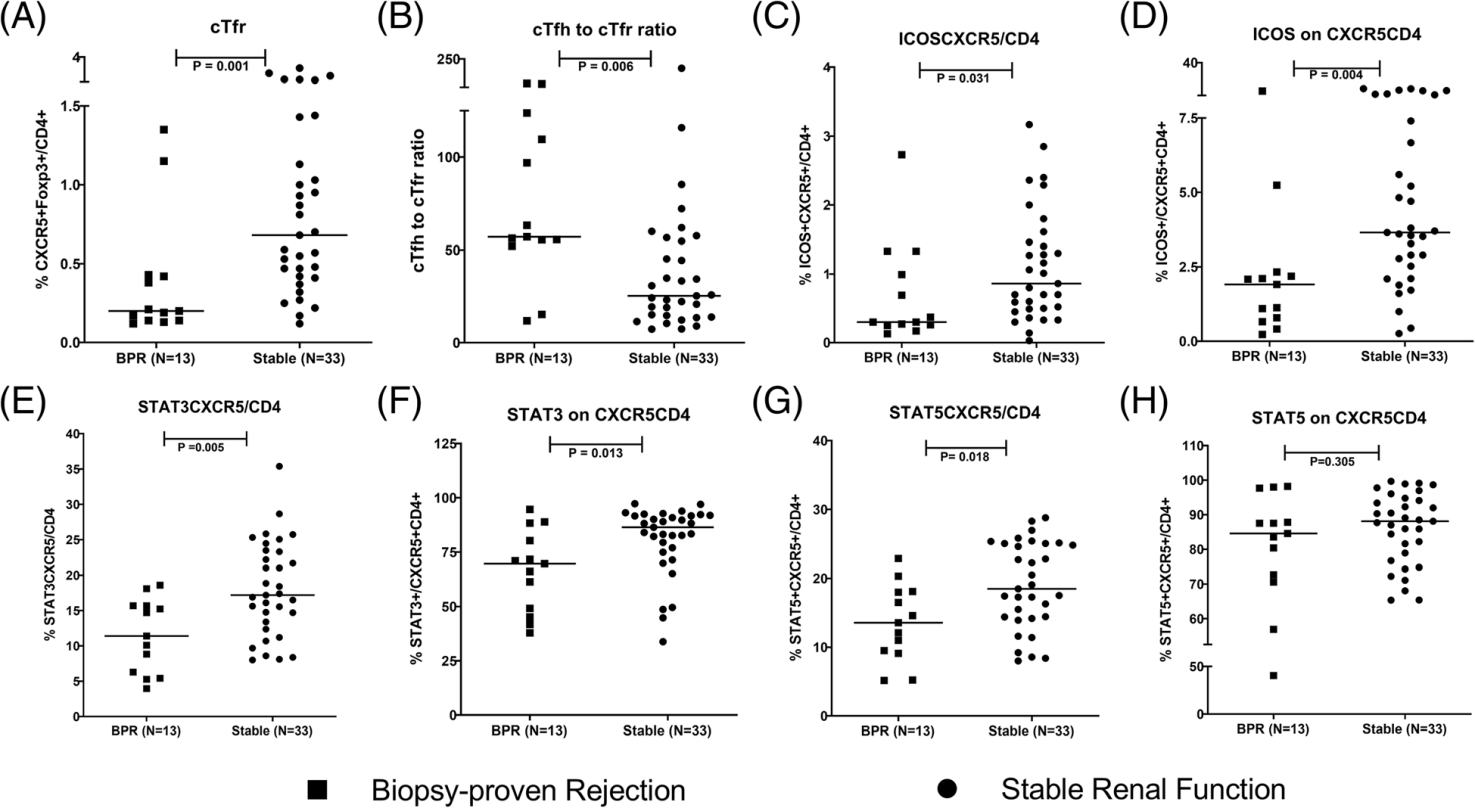
The frequency of cTfr cells, cTfh to cTfr ratio and the expression of ICOS, STAT3, STAT5 between BPR group and stable group. Squares refer to biopsy-proven rejection (BPR) group, cycles refer to stable renal function group; **a** cTfr: CXCR5^+^Foxp3^+^ on CD4^+^ cells; **b** cTfh to cTfr ratio; **c** CXCR5^+^ICOS^+^ on CD4^+^ cells; **d** ICOS^+^ on CXCR5^+^CD4^+^; **e** CXCR5^+^STAT3^+^ on CD4^+^ cells; **f** STAT3^+^ on CXCR5^+^CD4^+^ cells; **g** CXCR5^+^STAT5^+^ on CD4^+^ cells; **h** STAT5^+^ on CXCR5^+^CD4^+^ cells

**Fig. 4:**
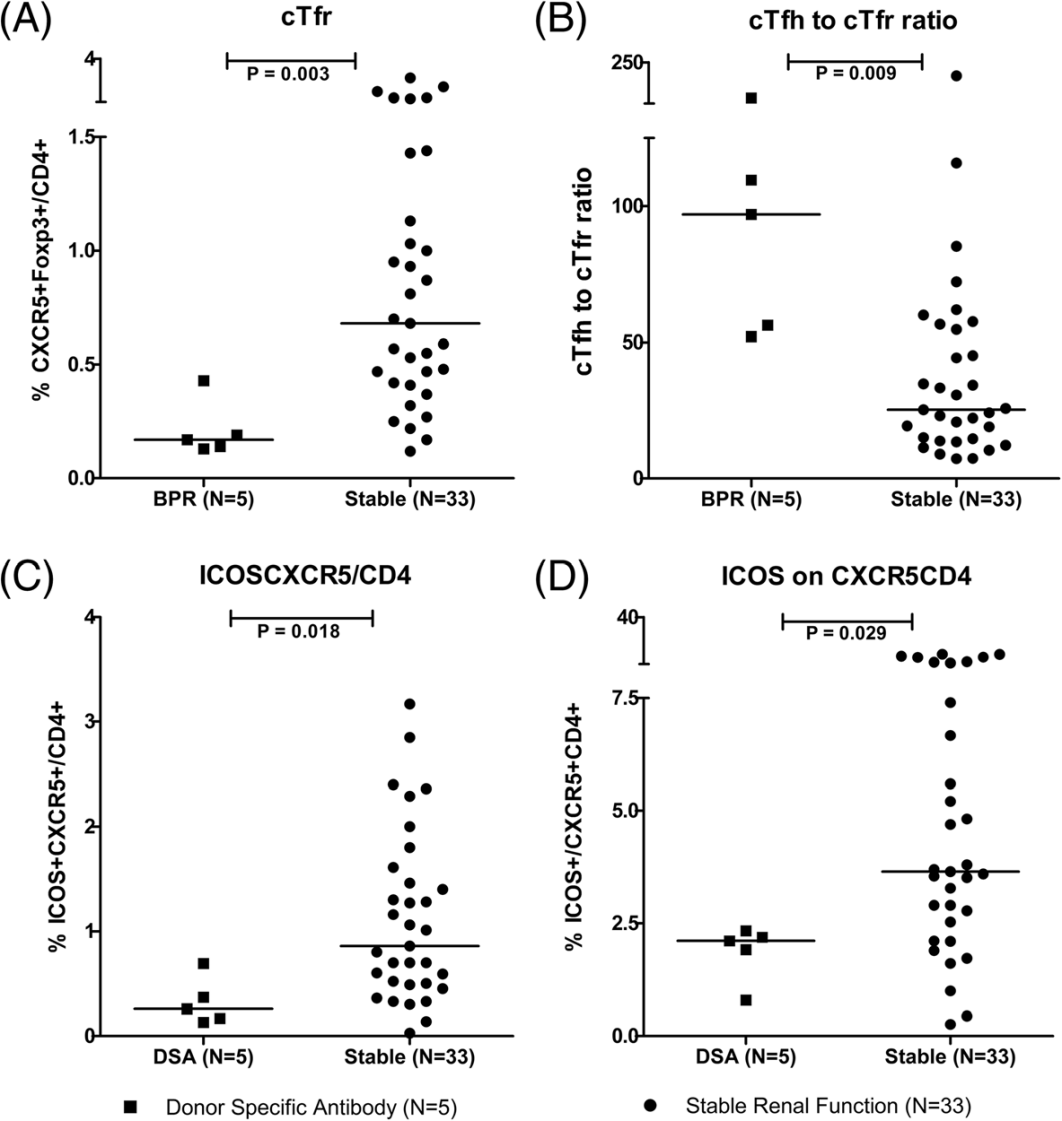
The frequency of cTfr cells, cTfh to cTfr ratio and the expression of ICOS between DSA positive group and stable group. Squares refer to donor specific antibody (DSA) group, cycles refer to stable renal function group; **a** cTfr: CXCR5^+^Foxp3^+^ on CD4^+^ cells; **b** cTfh to cTfr ratio; **c** CXCR5^+^ICOS^+^ on CD4^+^ cells; **d** ICOS^+^ on CXCR5^+^CD4^+^

When immune parameters were compared between BPR group and stable group, the percentage of cTfr, cTfh to cTfr ratio, the expression of ICOS, STAT3, STAT5 were significantly different (Fig. 3). The differences of other immune parameters were not significant (shown in Additional file 2). The percentage of cTfr, cTfh to cTfr ratio and ICOS expression was also significantly different between DSA positive group and stable group (Fig. 4**,** Additional file 3). The comparisons between non-rejection group and stable group, between non-rejection group and BPR group were also done. Only the percentage of CXCR5^+^STAT3^+^ on CD4^+^ was found significantly different between non-rejection group and stable group (shown in Additional file 4). Only cTfh to cTfr ratio and ICOS expression were found significantly different between BPR group and non-rejection group (shown in Additional file 5). The analysis results between PRA positive group and PRA negative group had the same trend as comparison between CAD group and stable group (shown in Additional file 6). Patients with CAD (eGFR< 60 ml/min/1.73m^2^) were divided into three groups: Group 1 with eGFR from 30 to 60 ml/min/1.73m^2^ (*N* = 19); Group 2 with eGFR from 15 to 30 ml/min/1.73m^2^ (*N* = 12); Group 3 with eGFR less than 15 ml/min/1.73m^2^ (*N* = 3). No significant differences of immune parameters were observed between these three groups (shown in Additional file 7).

## Discussion

In the present study, we found that the frequency of CXCR5^+^ on CD4^+^ cells and cTfr cells were decreased in CAD group than stable group. The frequency of cTfh cells had the same trend. The cTfh to cTfr ratio in CAD group was higher than that in stable group. Serum CXCL13 in CAD group was higher than that in stable group. Serum CXCL13 was negatively associated with the frequency of cTfh cells. No association between serum CXCL13 and cTfr cells was observed. Serum TGF-β in CAD group was lower than that in stable group. No association between serum TGF-β and cTfh, cTfr, or CXCR5^+^STAT3^+^ on CD4^+^cells was observed. The cTfh to cTfr ratio was an independent risk factor to renal function and CAD after multiple regression analysis. After stratified analysis based on the cTfh to cTfr ratio, the percentage of recipients with CAD in Group 4 was significantly higher than that in Group 1 and Group 2. The cTfh to cTfr ratio was also significantly higher in BPR group or DSA group compared to stable group.

The proportions of both cTfh and cTfr cells in recipients with CAD were lower than that in recipients with stable renal function. Tfh and Tfr cells share a lot of common differentiation signal pathway. Bcl-6 is a key transcription factor for the differentiation of Tfh and Tfr cells [6, 13]. Tfh and Tfr cells express CXCR5 and migrate into GC under the gradient of CXCL13 [6, 13]. Interleukin-2 (IL-2) could inhibit the differentiation of Tfh and Tfr cells through STAT5-Blimp-1 signal pathway [21–23]. A recent study demonstrated that RNA-binding protein (Roquin) could inhibit the differentiation from Naïve T cells to Tfh cells, while inhibit the conversion of Treg to Tfr cells through inhibiting protein kinase B signal pathway [24]. Several studies have demonstrated that STAT3 was indispensable for Tfh and Tfr cell differentiation by inducing the expression of Bcl-6 during immunization or infection [9, 10, 25, 26]. Therefore, it is probably that the increased number of Tfh cells would be accompanied with the increase of Tfr cells in kidney transplant recipients.

We found that serum CXCL13 in CAD group was significantly higher than that in stable group and high expression of serum CXCL13 is negatively associated with the frequency of cTfh cells. Havenar-Daughton et al. demonstrated in mouse, macaques model and HIV-infected human that plasma CXCL13 levels correlated with GC activity in draining lymph nodes [27]. Mabuka et al. found that early serum CXCL13 but not B cell frequency could predict the later emergence of detectable HIV neutralizing antibodies [28]. cTfh cells could migrate into GC via CXCL13 gradient, which might contribute to the lower frequency of cTfh cells in CAD group, initiate the GC formation, and promote the humoral immune response. Dedeoglu et al. recently demonstrated that the frequency of CD4^+^ T cells within CD3^+^ T cells in lymph node from end-stage renal disease (ESRD) patients was significantly higher than that in peripheral blood [29]. Tfh or Tfr cells, as a main subset of CD4^+^ T cells within GC, their frequency in GC was also probably higher than that in circulation, particularly under the chemotaxis of high level of CXCL13.

The cTfh to cTfr ratio was an independent risk factor to renal function and CAD after multiple regression analysis. The proportion of Tfh and Tfr cells is dramatically changed. In skin-draining lymph nodes without antigen stimulation, Tfr cells constitute approximately 50% of all CD4^+^CXCR5^+^ cells. Seven days after stimulation, Tfr cells comprised only approximately 20% of the CD4^+^CXCR5^+^ population [13]. When there was influenza infection, Tfr cells was approximately 5–8% of CD4^+^CXCR5^+^ cells [13]. Constant allograft antigen stimulation would make the imbalance between Tfh cells to Tfr cells, dysregulation of humoral immunity, and eventually lead to allograft rejection.

After stratified analysis based on the cTfh to cTfr ratio, the percentage of recipients with CAD in Group 4 was significantly higher than that in Group 1 and Group 2. The Tfh to Tfr ratio has been demonstrated to act as a biomarker of humoral immunity. Fan et al. recently demonstrated in a simian immunodeficiency virus-infected (SIV) rhesus macaques model that the Tfh to Tfr ratio in peripheral lymphatic tissues is critical for regulating autoreactive antibody production in chronic SIV infection [30]. Xu et al. found that the cTfh to cTfr ratio was associated with disease activity in systemic lupus erythematosus [25]. The cTfh to cTfr ratio indicated ectopic lymphoid structure formation in minor salivary gland, strongly associated with B cell, CD4^+^ T cell, and PD-1^+^ICOS^+^ T cell infiltration in minor salivary gland and allowed discrimination between Sjogren’s syndrome patients and healthy donors [31]. Chen et al. showed that the frequency of cTfr cells and the number of Tfr cells in renal graft tissues in ABMR group were significantly lower than that of non-ABMR group, although no cTfh to cTfr ratio was analyzed [15]. In the present study, we found that the cTfh to cTfr ratio was significantly higher in recipients with ABMR or DSA than recipients with stable renal function. The cTfh to cTfr ratio is a potential biomarker for kidney transplant recipients with CAD, ABMR and the production of DSA and might identify recipients at the risk of allograft failure.

No association between serum TGF-β level and cTfh cells nor CXCR5^+^STAT3^+^ on CD4^+^ T cells was observed. Serum TGF-β has a trend to be positively associated with cTfr cells. As it has been reported, TGF-β could not only contribute the Tfh differentiation but also Tfr production [8, 15, 16]. Tfh and Tfr cells are activated in germinal canter, which might explain why serum TGF-β did not significantly affect the frequency of cTfh and cTfr cells in kidney transplant recipients.

This study has some limitations. Only the percentage of each cell subset was detected, but not the cell function. The analysis of donor-specific immune cell would be better than the cell phenotyping for organ transplantation. In our previous study [32], we already found that donor-specific IL-21 producing cells at 6 months after kidney transplantation could predict rejection within 5 years, while cTfh or cTfr cells was not found to be associated with rejection. In the present study, cTfr cells and cTfh to cTfr ratio correlated with rejection, but not IL-21. Donor-specific IL-21 producing cells might be more sensitive in rejection prediction than cTfh and cTfr. However, with the prolonged transplant duration time, the expression of cTfh and cTfr might be changed. The different degree of CXCR5^+^ cells migrating to GC and allograft might lead to different outcomes. Considering the detection difficulty of donor-specific IL-21 producing cells, the cTfh to cTfr ratio might be more potential as a biomarker of CAD in kidney transplant recipients.

## Conclusion

The circulating Tfh to Tfr ratio was an independent risk factor for recipients with chronic renal allograft dysfunction. Serum CXCL13 level was negatively correlated with cTfh cells. Whether the cTfh to cTfr ratio and CXCL13 could predict the risk of CAD in kidney transplant recipients requires to be further clarified.

## Additional files

Additional file 1: **Figure S1.** Gating strategy used for the analysis of all immune parameters**.** (A) CXCR5 and Foxp3 Gating Strategy; (B) CXCR5 and PD-1 Gating Strategy; (C) CXCR5 and ICOS Gating Strategy; (D) CXCR5 and STAT3 Gating Strategy; (E) CXCR5 and STAT4 Gating Strategy; (F) CXCR5 and STAT5 Gating Strategy; (G) CXCR5 and IL-21 Gating Strategy. (JPG 3945 kb)

Additional file 2: **Figure S2.** The frequency of cTfh cells and the level of associated-factor between BPR group and stable group**.** Squares refer to biopsy-proven rejection (BPR) group, cycles refer to stable renal function group; (A) cTfh: CXCR5^+^Foxp3^−^ on CD4^+^ cells; (B) Tregs: CD25^+^Foxp3^+^ on CD4^+^ cells; (C) CXCR5^+^PD-1^+^ on CD4^+^ cells; (D) PD-1^+^ on CXCR5^+^CD4^+^ cells; (E) CXCR5^+^STAT4^+^ on CD4^+^ cells; (F) STAT4^+^ on CXCR5^+^CD4^+^ cells; (G) CXCR5^+^IL-21^+^ on CD4^+^ cells; (H) IL-21^+^ on CXCR5^+^CD4^+^ cells; (I) The serum level of CXCL13; (J) The serum level of TGF-β. (JPG 1525 kb)

Additional file 3: **Table S1.** Mann-Whitney U analysis between recipients with DSA and stable renal function**.** *P* < 0.05 were shown in bold. (DOCX 15 kb)

Additional file 4: **Table S2.** Mann-Whitney U analysis between recipients with stable renal function and non-rejection. *P* < 0.05 were shown in bold. (DOCX 15 kb)

Additional file 5: **Table S3.** Mann-Whitney U analysis between recipients with biopsy-proven rejection and non-rejection. *P* < 0.05 were shown in bold. (DOCX 15 kb)

Additional file 6: **Figure S3.** The frequency of cTfh and cTfr cells and the level of associated-factor between PRA positive group and PRA negative group. Squares refer to panel reactive antibodies positive (PRA) group, cycles refer to panel reactive antibodies negative group; (A) cTfh: CXCR5^+^Foxp3^−^ on CD4^+^ cells; (B) cTfr: CXCR5^+^Foxp3^+^ on CD4^+^ cells; (C) cTfh to cTfr ratio; (D) Tregs: CD25^+^Foxp3^+^ on CD4^+^ cells; (E) CXCR5^+^PD-1^+^ on CD4^+^ cells; (F) PD-1^+^ on CXCR5^+^CD4^+^ cells; (G) CXCR5^+^ICOS^+^ on CD4^+^ cells; (H) ICOS^+^ on CXCR5^+^CD4^+^; (I) CXCR5^+^STAT3^+^ on CD4^+^ cells; (J) STAT3^+^ on CXCR5^+^CD4^+^ cells; (K) CXCR5^+^STAT4^+^ on CD4^+^ cells; (L) STAT4^+^ on CXCR5^+^CD4^+^ cells; (M) CXCR5^+^STAT5^+^ on CD4^+^ cells; (N) STAT5^+^ on CXCR5^+^CD4^+^ cells; (O) CXCR5^+^IL-21^+^ on CD4^+^ cells; (P) IL-21^+^ on CXCR5^+^CD4^+^ cells; (Q) The serum level of CXCL13; (R) The serum level of TGF-β. (JPG 2686 kb)

Additional file 7: **Table S4.** Kruskal-Wallis H analysis of immune parameters between three groups of recipients with CAD divided by eGFR. Thirty-four patients with CAD (eGFR< 60 ml/min/1.73m^2^) were divided into three groups: Group 1 with eGFR from 30 to 60 ml/min/1.73m^2^ (*N* = 19); Group 2 with eGFR from 15 to 30 ml/min/1.73m^2^ (*N* = 12); Group 3 with eGFR less than 15 ml/min/1.73m^2^ (*N* = 3). (DOCX 15 kb)

## Data Availability

The datasets used in the current study are available from the corresponding author on reasonable request.

## Abbreviations

ABMR: Antibody-mediated rejection
Bcl-6: B cell lymphoma 6
Blimp-1: B lymphocyte-induced maturation protein 1
BPR: Biopsy-proven rejection
CAD: Chronic renal allograft dysfunction
CNI: Calcineurin inhibitor
cTfh: Circulating Tfh
cTfr: Circulating Tfr
CXCL13: C-X-C chemokine ligand 3
CXCR5: C-X-C chemokine receptor 5
DSA: Donor-specific antibodies
eGFR: Estimated glomerular filtration rate
ESRD: End-stage renal disease
FoxP3: Foxkhead box P3
GC: Germinal center
ICOS: Inducible co-stimulator
MMF: Mycophenolate mofetil
PD-1: Programmed death 1
PMA: Phorbol 12-myristate 13-acetate
PRA: Panel reactive antibodies
Scr: Serum creatinine
SIV: Simian immunodeficiency virus
STAT3: Signal transducer and activator of transcription 3
STAT5: Signal transducer and activator of transcription 5
TCMR: T cell-mediated rejection
Tfh cells: T follicular helper cells
Tfr cells: T follicular regulatory cells
TGF-β: Transforming growth factor beta
Tregs: Regulatory T cells
