# Supplementary figures and images for: Increased circulating Tfh to Tfr ratio in chronic renal allograft dysfunction: a pilot study
Source: BMC Immunol. 2019 Aug 5;20:26. doi: 10.1186/s12865-019-0308-x (PMC6683539; doi:10.1186/s12865-019-0308-x)

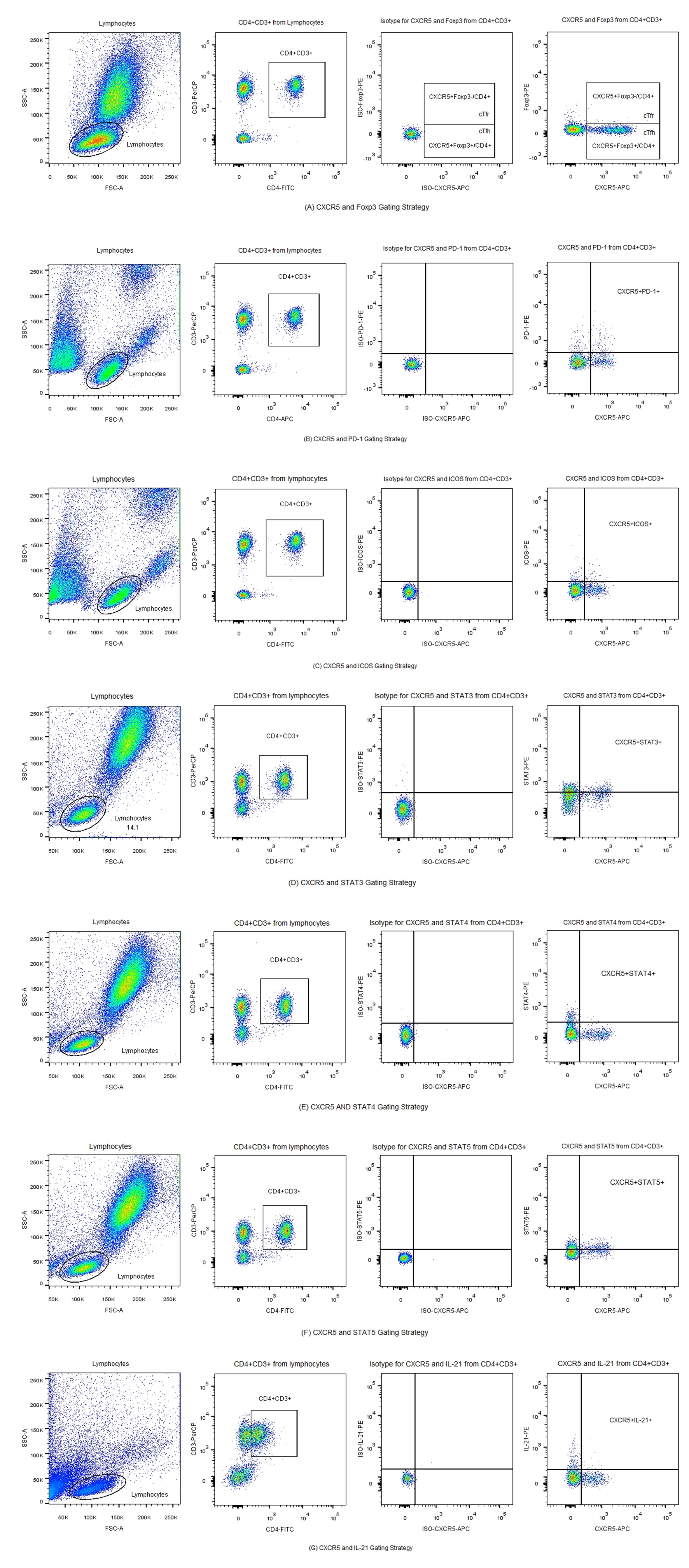

Supplement: Supplementary file 1 — Figure S1. Gating strategy used for the analysis of all immune parameters. (A) CXCR5 and Foxp3 Gating Strategy; (B) CXCR5 and PD-1 Gating Strategy; (C) CXCR5 and ICOS Gating Strategy; (D) CXCR5 and STAT3 Gating Strategy; (E) CXCR5 and STAT4 Gating Strategy; (F) CXCR5 and STAT5 Gating Strategy; (G) CXCR5 and IL-21 Gating Strategy. (JPG 3945 kb) [file 12865_2019_308_MOESM1_ESM.jpg]

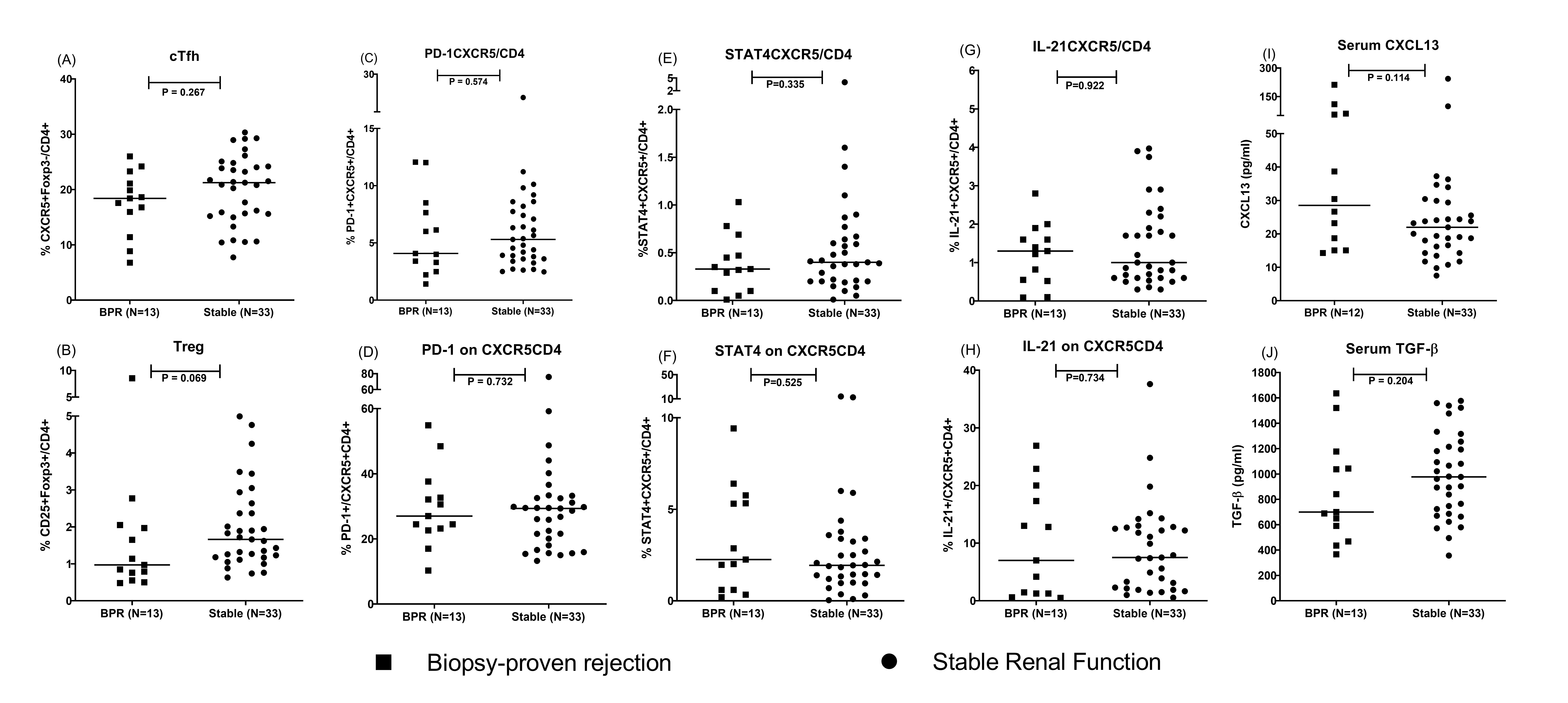

Supplement: Supplementary file 2 — Figure S2. The frequency of cTfh cells and the level of associated-factor between BPR group and stable group. Squares refer to biopsy-proven rejection (BPR) group, cycles refer to stable renal function group; (A) cTfh: CXCR5+Foxp3− on CD4+ cells; (B) Tregs: CD25+Foxp3+ on CD4+ cells; (C) CXCR5+PD-1+ on CD4+ cells; (D) PD-1+ on CXCR5+CD4+ cells; (E) CXCR5+STAT4+ on CD4+ cells; (F) STAT4+ on CXCR5+CD4+ cells; (G) CXCR5+IL-21+ on CD4+ cells; (H) IL-21+ on CXCR5+CD4+ cells; (I) The serum level of CXCL13; (J) The serum level of TGF-β. (JPG 1525 kb) [file 12865_2019_308_MOESM2_ESM.jpg]
