# Supplementary material for: Increased circulating Tfh to Tfr ratio in chronic renal allograft dysfunction: a pilot study
Source: BMC Immunol. 2019 Aug 5;20:26. doi: 10.1186/s12865-019-0308-x (PMC6683539; doi:10.1186/s12865-019-0308-x)
Supplement: Supplementary file 3 — Table S1. Mann-Whitney U analysis between recipients with DSA and stable renal function. P < 0.05 were shown in bold. (DOCX 15 kb) [file 12865_2019_308_MOESM3_ESM.docx]

**Table S1. Mann-Whitney U analysis between recipients with DSA and stable renal function**

| Test Statistics^a^ | | | | | | | | | | | | | | | | | | | | |
| --- | --- | --- | --- | --- | --- | --- | --- | --- | --- | --- | --- | --- | --- | --- | --- | --- | --- | --- | --- | --- |
|  | CXCR5 | TFH | TFR | RATIO | TREG | PD1CXCR5 | PD1ONCXCR5 | ICOSCXCR5 | ICOSONCXCR5 | STAT3CXCR5 | STAT3ONCXCR5 | STAT4CXCR5 | STAT4ONCXCR5 | STAT5CXCR5 | STAT5ONCXCR5 | IL21CXCR5 | IL21ONCXCR5 | CXCL13 | TGFB |  |
| Mann-Whitney U | 62.0 | 63.5 | 14.5 | 22.0 | 60.0 | 82.0 | 60.0 | 23.0 | 31.5 | 45.5 | 72.0 | 79.5 | 63.0 | 52.0 | 56.0 | 79.0 | 58.0 | 62.5 | 73.0 |  |
| Wilcoxon W | 77.0 | 78.5 | 29.5 | 583.0 | 75.0 | 643.0 | 621.0 | 38.0 | 46.5 | 60.5 | 87.0 | 640.5 | 624.0 | 67.0 | 71.0 | 640.0 | 619.0 | 558.5 | 88.0 |  |
| Z | -.885 | -.821 | -2.937 | -2.613 | -.972 | -.022 | -.972 | -2.570 | -2.203 | -1.598 | -.453 | -.130 | -.842 | -1.317 | -1.144 | -.151 | -1.058 | -.687 | -.311 |  |
| Asymp. Sig. (2-tailed) | .376 | .412 | **.003** | **.009** | .331 | .983 | .331 | **.010** | **.028** | .110 | .650 | .897 | .400 | .188 | .252 | .880 | .290 | .492 | .756 |  |
| Exact Sig. [2*(1-tailed Sig.)] | .399^b^ | .424^b^ | **.001^b^** | **.006^b^** | .353^b^ | 1.000^b^ | .353^b^ | **.008^b^** | **.024^b^** | .112^b^ | .675^b^ | .900^b^ | .424^b^ | .202^b^ | .271^b^ | .900^b^ | .310^b^ | .504^b^ | .780^b^ |  |
| a. Grouping Variable: DSA vs Stable | | | | | | | | | | | | | | | | | | | | |
| b. Not corrected for ties. | | | | | | | | | | | | | | | | | | | | |

P<0.05 were shown in bold
