# Supplementary material for: Increased circulating Tfh to Tfr ratio in chronic renal allograft dysfunction: a pilot study
Source: BMC Immunol. 2019 Aug 5;20:26. doi: 10.1186/s12865-019-0308-x (PMC6683539; doi:10.1186/s12865-019-0308-x)
Supplement: Supplementary file 4 — Table S2. Mann-Whitney U analysis between recipients with stable renal function and non-rejection. P < 0.05 were shown in bold. (DOCX 15 kb) [file 12865_2019_308_MOESM4_ESM.docx]

**Table S2. Mann-Whitney U analysis between recipients with stable renal function and non-rejection**

| Test Statistics^a^ | | | | | | | | | | | | | | | | | | | |
| --- | --- | --- | --- | --- | --- | --- | --- | --- | --- | --- | --- | --- | --- | --- | --- | --- | --- | --- | --- |
|  | CXCR5 | TFH | TFR | RATIO | TREG | PD1CXCR5 | PD1ONCXCR5 | ICOSCXCR5 | ICOSONCXCR5 | STAT3CXCR5 | STAT3ONCXCR5 | STAT4CXCR5 | STAT4ONCXCR5 | STAT5CXCR5 | STAT5ONCXCR5 | IL21CXCR5 | IL21ONCXCR5 | CXCL13 | TGFB |
| Mann-Whitney U | 109.0 | 114.0 | 131.5 | 150.5 | 132.5 | 144.5 | 119.5 | 156.5 | 129.0 | 79.5 | 131.0 | 141.5 | 151.0 | 53.5 | 107.0 | 151.0 | 163.5 | 44.5 | 26.0 |
| Wilcoxon W | 164.0 | 169.0 | 186.5 | 711.5 | 187.5 | 199.5 | 680.5 | 717.5 | 690.0 | 134.5 | 186.0 | 196.5 | 712.0 | 108.5 | 162.0 | 206.0 | 724.5 | 50.5 | 32.0 |
| Z | -1.610 | -1.466 | -.963 | -.417 | -.934 | -.589 | -1.308 | -.244 | -1.035 | -2.458 | -.977 | -.676 | -.403 | -3.206 | -1.668 | -.403 | -.043 | -.121 | -1.296 |
| Asymp. Sig. (2-tailed) | .107 | .143 | .335 | .677 | .350 | .556 | .191 | .807 | .301 | **.014** | .328 | .499 | .687 | .001 | .095 | .687 | .966 | .903 | .195 |
| Exact Sig. [2*(1-tailed Sig.)] | .112^b^ | .149^b^ | .341^b^ | .681^b^ | .356^b^ | .561^b^ | .194^b^ | .810^b^ | .313^b^ | **.012^b^** | .341^b^ | .505^b^ | .702^b^ | .001^b^ | .099^b^ | .702^b^ | .966^b^ | .909^b^ | .217^b^ |
| a. Grouping Variable: Stable vs Non-rejection | | | | | | | | | | | | | | | | | | | |
| b. Not corrected for ties. | | | | | | | | | | | | | | | | | | | |

P<0.05 were shown in bold
