# Supplementary material for: Increased circulating Tfh to Tfr ratio in chronic renal allograft dysfunction: a pilot study
Source: BMC Immunol. 2019 Aug 5;20:26. doi: 10.1186/s12865-019-0308-x (PMC6683539; doi:10.1186/s12865-019-0308-x)
Supplement: Supplementary file 5 — Table S3. Mann-Whitney U analysis between recipients with biopsy-proven rejection and non-rejection. P < 0.05 were shown in bold. (DOCX 15 kb) [file 12865_2019_308_MOESM5_ESM.docx]

**Table S3. Mann-Whitney U analysis between recipients with biopsy-proven rejection and non-rejection**

| Test Statistics^a^ | | | | | | | | | | | | | | | | | | | | |
| --- | --- | --- | --- | --- | --- | --- | --- | --- | --- | --- | --- | --- | --- | --- | --- | --- | --- | --- | --- | --- |
|  | CXCR5 | TFH | TFR | RATIO | TREG | PD1CXCR5 | PD1ONCXCR5 | ICOSCXCR5 | ICOSONCXCR5 | STAT3CXCR5 | STAT3ONCXCR5 | STAT4CXCR5 | STAT4ONCXCR5 | STAT5CXCR5 | STAT5ONCXCR5 | IL21CXCR5 | IL21ONCXCR5 | CXCL13 | TGFB |  |
| Mann-Whitney U | 55.5 | 57.0 | 37.0 | 29.0 | 60.0 | 63.0 | 46.0 | 31.5 | 21.0 | 64.5 | 46.0 | 63.0 | 62.5 | 48.5 | 56.0 | 62.5 | 58.0 | 15.0 | 16.0 |  |
| Wilcoxon W | 110.5 | 112.0 | 128.0 | 84.0 | 151.0 | 154.0 | 137.0 | 122.5 | 112.0 | 155.5 | 137.0 | 118.0 | 153.5 | 103.5 | 111.0 | 153.5 | 149.0 | 21.0 | 22.0 |  |
| Z | -.589 | -.496 | -1.737 | -2.233 | -.310 | -.124 | -1.179 | -2.079 | -2.729 | -.031 | -1.178 | -.124 | -.155 | -1.024 | -.558 | -.155 | -.434 | -.433 | -.471 |  |
| Asymp. Sig. (2-tailed) | .556 | .620 | .082 | **.026** | .756 | .901 | .239 | **.038** | **.006** | .975 | .239 | .901 | .877 | .306 | .577 | .877 | .664 | .665 | .638 |  |
| Exact Sig. [2*(1-tailed Sig.)] | .563^b^ | .648^b^ | .088^b^ | **.026^b^** | .784^b^ | .927^b^ | .257^b^ | **.036^b^** | **.005^b^** | .976^b^ | .257^b^ | .927^b^ | .879^b^ | .313^b^ | .605^b^ | .879^b^ | .693^b^ | .734^b^ | .704^b^ |  |
| a. Grouping Variable: BPR VS Non-rejection | | | | | | | | | | | | | | | | | | | | |

P<0.05 were shown in bold
