# Supplementary material for: Increased circulating Tfh to Tfr ratio in chronic renal allograft dysfunction: a pilot study
Source: BMC Immunol. 2019 Aug 5;20:26. doi: 10.1186/s12865-019-0308-x (PMC6683539; doi:10.1186/s12865-019-0308-x)
Supplement: Supplementary file 7 — Table S4. Kruskal-Wallis H analysis of immune parameters between three groups of recipients with CAD divided by eGFR. Thirty-four patients with CAD (eGFR< 60 ml/min/1.73m2) were divided into three groups: Group 1 with eGFR from 30 to 60 ml/min/1.73m2 (N = 19); Group 2 with eGFR from 15 to 30 ml/min/1.73m2 (N = 12); Group 3 with eGFR less than 15 ml/min/1.73m2 (N = 3). (DOCX 15 kb) [file 12865_2019_308_MOESM7_ESM.docx]

| **Test Statistics^a,b^** | | | | | | | | | | | | | | | | | | | | |
| --- | --- | --- | --- | --- | --- | --- | --- | --- | --- | --- | --- | --- | --- | --- | --- | --- | --- | --- | --- | --- |
|  | **CXCR5** | **TFH** | **TFR** | **RATIO** | **TREG** | **PD1CXCR5** | **PD1ONCXCR5** | **ICOSCXCR5** | **ICOSONCXCR5** | **STAT3CXCR5** | **STAT3ONCXCR5** | **STAT4CXCR5** | **STAT4ONCXCR5** | **STAT5CXCR5** | **STAT5ONCXCR5** | **IL21CXCR5** | **IL21ONCXCR5** | **CXCL13** | **TGFB** |  |
| **Chi-Square** | **.931** | **.851** | **.537** | **.045** | **.942** | **.637** | **.314** | **.441** | **1.797** | **1.753** | **3.316** | **2.088** | **1.874** | **.033** | **1.966** | **1.852** | **1.430** | **1.772** | **.535** |  |
| **df** | **2** | **2** | **2** | **2** | **2** | **2** | **2** | **2** | **2** | **2** | **2** | **2** | **2** | **2** | **2** | **2** | **2** | **2** | **2** |  |
| **Asymp. Sig.** | **.628** | **.653** | **.765** | **.978** | **.624** | **.727** | **.855** | **.802** | **.407** | **.416** | **.191** | **.352** | **.392** | **.984** | **.374** | **.396** | **.489** | **.412** | **.765** |  |
| **a. Kruskal Wallis Test** | | | | | | | | | | | | | | | | | | | | |
| **b. Grouping Variable: subCAD** | | | | | | | | | | | | | | | | | | | | |

**Table S4. Kruskal-Wallis H analysis of immune parameters between three groups of recipients with CAD divided by eGFR.**

34 patients with CAD (eGFR<60 ml/min/1.73m^2^) were divided into three groups: Group 1 with eGFR from 30 to 60 ml/min/1.73m^2^ (N=19); Group 2 with eGFR from 15 to 30 ml/min/1.73m^2^ (N=12); Group 3 with eGFR less than 15 ml/min/1.73m^2^ (N=3).
